# Glyphosate exposure and hormonal disruption in pregnancy: evidence from a birth cohort in Puerto Rico

**DOI:** 10.1038/s41370-026-00902-6

**Published:** 2026-06-15

**Authors:** Mislael A. Valentín-Cortés, Amber L. Cathey, Haley M. Jenkins, Jennifer Fernandez, Zaira Rosario Pabón, Carmen M. Vélez Vega, José F. Cordero, Akram Alshawabkeh, Deborah J. Watkins, John D. Meeker

**Affiliations:** 1 Department of Epidemiology, University of Michigan School of Public Health, Ann Arbor, MI USA; 2 Department of Environmental Health Sciences, University of Michigan, School of Public Health, Ann Arbor, MI USA; 3 University of Puerto Rico Medical Sciences Campus, Graduate School of Public Heth, San Juan, PR USA; 4 Department of Epidemiology and Biostatistics, University of Georgia, Athens, GA USA; 5 Department of Civil and Environmental Engineering, Northeastern University, Boston, MA USA

**Keywords:** Glyphosate, Endocrine Disruption, Pregnancy Hormones, Aminomethylphosphonic Acid, Prenatal Exposure

## Abstract

**Background:**

Glyphosate and its primary metabolite, aminomethylphosphonic acid (AMPA), are widespread environmental contaminants with potential endocrine-disrupting properties. Despite near-universal glyphosate and AMPA exposure, human studies evaluating their association with maternal hormonal regulation during gestation remain scarce.

**Objective:**

To assess associations between urinary glyphosate and AMPA concentrations and repeated measures of maternal hormone levels during pregnancy and explore effect modification by gestational timing and fetal sex.

**Methods:**

We analyzed data from 752 pregnancies in the PROTECT birth cohort in Puerto Rico. Glyphosate and AMPA were measured in up to three urine samples per participant collected at 18 ± 2-, 22 ± 2-, and 26 ± 2-weeks’ gestation. Serum concentrations of sex steroid (estriol, progesterone, testosterone), thyroid (T3, T4, fT4, TSH), and stress-related (CRH) hormones were assessed at two periods. Linear mixed effects models estimated differences in serum hormone levels associated with interquartile range (IQR) increases in urinary glyphosate/AMPA, adjusting for demographic, biological, and technical covariates.

**Results:**

Glyphosate and AMPA were associated with lower estriol concentrations across pregnancy. Each IQR increase in AMPA was associated with 10.6% lower estriol (95% CI: –18.1%, –2.4%), while glyphosate was associated with 8.3% lower estriol (95% CI: –14.6%, –1.4%). AMPA was associated with 4.0% higher T3 (95% CI: 0.9%, 7.0%). In sensitivity analyses, AMPA was linked to 7.9% higher TSH at 26 ± 2-weeks’ gestation (95% CI: 0.5%, 15.9%), while glyphosate was associated with 9.5% higher CRH at this time point (95% CI: 1.5%, 18.2%). Stratified models revealed stronger associations among participants carrying female versus male fetuses.

**Significance:**

This study provides first evidence of associations between prenatal glyphosate and AMPA exposure and disruption of multiple maternal hormonal pathways during pregnancy. Findings suggest these contaminants may interfere with estrogenic, thyroid, and stress hormone systems critical for pregnancy maintenance and fetal development, with implications for understanding mechanisms underlying adverse birth outcomes.

**Impact:**

This study provides the first human evidence that glyphosate and AMPA, ubiquitous environmental contaminants detected in 75–95% of pregnant women, disrupt multiple maternal hormonal pathways during pregnancy. Among 752 participants in a Puerto Rico birth cohort, we demonstrate significant reductions in estriol (8–11% decreases) and alterations in thyroid and stress hormones associated with glyphosate exposure. These findings illuminate potential biological mechanisms underlying previously observed associations between glyphosate exposure and adverse birth outcomes, including preterm birth and altered fetal growth. Given the widespread use of glyphosate-based herbicides globally, our results have significant implications for public health policy and regulatory decision-making. The hormonal disruptions we identified suggest that pregnant populations may be particularly vulnerable to glyphosate exposure, supporting the need for enhanced protective measures and exposure reduction strategies during critical windows of fetal development.

## Introduction

Glyphosate, a broad-spectrum systemic herbicide, is the most widely used agricultural chemical globally, with use in the United States alone increasing from 0.36 million kilograms in 1974 to over 36 million kilograms by 2000 and continued rise in subsequent years [1]. Glyphosate-based herbicides (GBHs), marketed under diverse name brands, are extensively applied across agricultural, residential, and industrial settings, contributing to widespread human exposure. Once introduced into the environment, glyphosate degrades to aminomethylphosphonic acid (AMPA), a stable and persistent compound that accumulates in soil, water, and food systems [2, 3]. Both glyphosate and AMPA have been detected in 75–95% of urine samples collected from pregnant individuals in diverse populations across North America, raising significant public health concerns, especially regarding exposure during critical windows of fetal development [4–9]. Human exposure occurs primarily through ingestion of contaminated food or water, inhalation of pesticide drift, and dermal contact in treated environments [10–14]. Populations living near agricultural zones and/or with limited regulatory protections may face disproportionately higher exposures due to increased proximity to pesticide applications, occupational exposure, or inadequate environmental monitoring [5, 15].

Recent toxicological and epidemiologic literature has highlighted the potential for glyphosate and GBHs to act as endocrine-disrupting chemicals (EDCs). This class of compounds interferes with hormone biosynthesis, receptor binding, or feedback regulation within the endocrine system [16, 17]. These disruptions can occur even at low levels of exposure and may not follow traditional dose-response patterns, particularly when exposure occurs during sensitive developmental windows [16, 17]. EDCs such as glyphosate are particularly concerning since they may affect multiple hormonal pathways simultaneously, potentially amplifying physiologic disruption during critical life stages.

Pregnancy is one such vulnerable period, since it is characterized by extensive hormonal changes that support maternal adaptation and fetal development. Key hormonal systems involved in pregnancy include the sex steroid hormone axis (e.g., estriol, progesterone, testosterone), the hypothalamic-pituitary-thyroid (HPT) axis (e.g., triiodothyronine [T3], thyroxine [T4], thyroid-stimulating hormone [TSH]), and the hypothalamic-pituitary-adrenal (HPA) axis (e.g., corticotropin-releasing hormone [CRH]). These hormonal pathways are essential for maintaining uterine quiescence, modulating placental function, guiding neurodevelopment, and regulating the timing of labor and delivery [17, 18]. Even modest disruptions to these systems during gestation may alter fetal growth trajectories, increase the risk of preterm birth, or have long-term consequences for offspring health [17–19].

In vitro studies suggest that glyphosate and GBHs may influence estrogen signaling and steroidogenesis, potentially via inhibition of aromatase, a key enzyme responsible for the conversion of androgens into estrogens [20]. Inhibition of aromatase could impair placental estriol synthesis, which plays a critical role in maintaining uterine blood flow and supporting fetal development [21, 22]. Animal studies replicate these findings, with perinatal exposures linked to reduced circulating estrogen levels, changes in the expression of estrogen and progesterone receptors, and disruption of the HPT axis [23–27]. Though less extensively studied, animal models suggest that glyphosate exposure may affect the HPA axis, potentially leading to upregulation of CRH, a key regulator for the timing of labor [28].

Collectively, these mechanistic studies support the plausibility of endocrine disruption via multiple hormonal pathways relevant to pregnancy, particularly those involving estriol, T3, and CRH, which were assessed in the present study. The observed effects vary by dose, formulation, and timing of exposure, underscoring the importance of characterizing real-world exposures and sensitive windows during human gestation. Despite this growing mechanistic evidence, human epidemiologic research on glyphosate’s endocrine-disrupting potential remains limited, especially in pregnancy. Parvez et al. found that higher urinary glyphosate levels were associated with shortened gestational length in a small cohort of pregnant women in Indiana [4]. Similarly, Gerona et al. reported associations between higher first-trimester glyphosate levels and reduced fetal growth in a racially diverse sample of high-risk pregnancies [15]. In the PROTECT cohort in Puerto Rico, Silver et al. observed that second-trimester urinary concentrations of glyphosate and AMPA were significantly associated with increased odds of preterm birth [5]. Together, these studies highlight consistent associations between glyphosate-related exposures and adverse birth outcomes but offer limited insight into underlying biological mechanisms. Notably, none of these studies assessed maternal hormone concentrations, despite widespread recognition of endocrine pathways as key mediators of gestational health. Furthermore, epidemiologic evidence for AMPA, a major glyphosate degradate detected in nearly all participants, remains particularly sparse.

To our knowledge, no human cohort study has directly assessed associations between urinary glyphosate or AMPA concentrations and longitudinal profiles of maternal hormone levels during pregnancy. This represents a critical knowledge gap, especially given the high prevalence of exposure and the central role of endocrine signaling in pregnancy maintenance, fetal development, and birth timing. Understanding how glyphosate and AMPA affect maternal hormone concentrations can clarify whether observed adverse outcomes (e.g., preterm birth, fetal growth restriction) might be mediated through disruptions in the sex steroid, thyroid, or HPA axes. The current study addresses this critical gap by examining repeated measures of urinary glyphosate and AMPA in relation to maternal hormone levels in a prospective birth cohort in Puerto Rico. Specifically, we draw on data from the PROTECT cohort, which includes a diverse, predominantly low-income population, with extensive longitudinal biomarker and demographic data. Our objectives are threefold: [1] to evaluate associations between glyphosate and AMPA concentrations and changes in maternal sex steroid, thyroid, and stress-related hormones across pregnancy; [2] to assess whether these associations vary by gestational timing, suggesting critical windows of susceptibility; and [3] to explore whether fetal sex modifies these associations, given emerging evidence of sex-specific endocrine responses.

## Methods

### Study population

Study protocols for recruitment into the PROTECT cohort have been described previously [19]. Briefly, women residing in the northern karst region of Puerto Rico were recruited from seven prenatal clinics and hospitals around 14 weeks gestation, and this analysis used data collected between 2011 and 2019. Women were eligible to participate if they were between the ages of 18 and 40 years, had not used in vitro fertilization to become pregnant, did not use oral contraceptives within 3 months of becoming pregnant, and did not have any major preexisting medical or obstetric conditions. All participants provided written informed consent, and the study protocols were approved by research ethics committees at all participating institutions.

### Assessment of glyphosate and AMPA exposure biomarkers

Details on the measurement of urinary glyphosate and AMPA concentrations as biomarkers of exposure to these compounds have been described previously [5]. Briefly, spot urine samples were collected from participants at up to three time points during pregnancy (approximately 18 ± 2-, 22 ± 2-, and 26 ± 2-weeks’ gestation). Samples were then frozen at -80°C and shipped on dry ice to be analyzed at NSF International in Ann Arbor, MI, using gas chromatography tandem triple quadrupole mass spectrometry (GC-MS/MS). Concentrations below the limit of detection (LOD) were imputed with the LOD divided by the square root of two. Specific gravity was measured at the University of Puerto Rico Medical Sciences Campus using a handheld digital refractometer (Atago Co., Ltd., Tokyo, Japan) to account for differences in urine dilution between samples.

### Assessment of hormone concentrations

Serum samples were collected from participants at the first and third study visits (roughly 18 ± 2- and 26 ± 2-weeks’ gestation). Samples were analyzed at the Central Ligand Assay Satellite Services laboratory in the Department of Epidemiology at the University of Michigan School of Public Health. Immunoassays were used to quantify progesterone, sex hormone binding globulin (SHBG), testosterone, total triiodothyronine (T3), total thyroxine (T4), free thyroxine (fT4), and thyroid stimulating hormone (TSH), corticotropin releasing hormone (CRH), and estriol (E3); details on these assays have been published previously [29].

Hormone data included in the present analysis were assayed in three different batches occurring in 2016, 2017, and 2019. The lab equipment used to quantify testosterone, SHBG, E3, progesterone, and all thyroid hormones changed between 2017 and 2019. Thus, all hormone measurements were assigned a method variable value to indicate whether they were measured in the first two batches (2016 and 2017) or the third batch (2019).

### Statistical analyses

Distributions of demographic and other potential covariates were tabulated, including means, counts, and percentiles. Distributions and summary statistics of visit-specific urinary glyphosate and AMPA concentrations and serum hormone concentrations were also calculated. For analyses using average glyphosate and AMPA concentrations across pregnancy, concentrations were corrected for specific gravity using the formula X_SG_ = X[(SG_m_–1)/(SG_i_–1)] where X_SG_ represents the corrected concentration, X represents the uncorrected concentration, SG_M_ represents the median specific gravity value within the study population, and SG_i_ represents the specific gravity value of a given participant [30]. Geometric means of glyphosate and AMPA concentrations corrected for specific gravity were calculated using all available measurements for each study participant to represent overall pregnancy exposure levels. Log-normal hormones were transformed prior to analysis (CRH, E3, progesterone, testosterone, and TSH).

Primary statistical analyses utilized linear mixed effects (LME) models to regress repeated measures of hormone concentrations on repeated measures of glyphosate and AMPA (not corrected for specific gravity). A random intercept was included in all models to account for within-participant correlations. Based on a priori knowledge, primary statistical models were adjusted for maternal age and pre-pregnancy BMI (continuous), maternal education and parity (categorical), fetal sex, and urinary specific gravity. Since hormone levels were measured in multiple batches spanning several years, we included a batch indicator variable in all models to account for potential batch effects. The final analytical dataset contained 752 participants for whom we had complete covariate data and at least one exposure biomarker and hormone measurement pair; only complete cases were included for analysis. In the sample, 303 participants provided one exposure measure, 274 participants provided two exposure measures, and 175 participants provided three exposure measures. Effect estimates are expressed as the percent change in hormone concentration per interquartile range (IQR) increase in glyphosate or AMPA concentration. We conducted a sensitivity analysis regressing visit-specific hormone concentrations on specific gravity-corrected geometric mean concentrations of individual participant glyphosate and AMPA across pregnancy to explore possible windows of susceptibility to hormone perturbation. An additional sensitivity analysis evaluated potential non-linearity of associations between GLY/AMPA and hormone concentrations by modeling GLY/AMPA as quartiles, with the lowest quartile as the reference group, in LME models. A secondary analysis explored fetal sex as an effect modifier in LME models. To achieve this, we utilized interaction terms between exposure variables and fetal sex indicator variables where the p-value of the interaction term can be interpreted as the significance level of effect modification, and the fetal sex indicator variable was defined such that the main exposure coefficient corresponded to the estimate among a particular fetal sex. All analyses were conducted in R version 4.4.1. Significance levels for all analyses were set to alpha = 0.05.

## Results

Distributions of demographic and other potential covariates are shown in Table 1. Participants had a median age of 27 years and a median pre-pregnancy BMI of 24.7 kg/m². Most women were married (52.7%), had attained at least some college education (57%), did not drink alcohol (94.2%) or smoke (98.1%) during pregnancy, lived in a household earning less than $30,000 per year (63.9%), were never exposed to environmental tobacco smoke (90.2%), were nulliparous (42.7%), and were carrying a male fetus (51.2%).

**Table 1:** Demographic, social, and health characteristics among 752 women in the PROTECT birth cohort in Puerto Rico, 2011–2019.

	*N* (%)		*N* (%)
Maternal age (years)		Annual household income	
18–24	279 (37.1%)	<10k	217 (32.9%)
25–29	231 (30.7%)	10k–<30k	204 (31.0%)
30–34	151 (20.1%)	30k–<50k	152 (23.1%)
35–41	91 (12.1%)	≥50k	86 (13.1%)
Missing	0	Missing	93
**Maternal Education**		**Smoking Status**	
GED or less	165 (21.9%)	Never	643 (85.5%)
Some college	264 (35.1%)	Ever	95 (12.6%)
Bachelors or higher	323 (43.0%)	Current	14 (1.9%)
Missing	0	Missing	0
**Marital Status**		**Environmental Tobacco Smoke Exposure**	
Single	149 (19.9%)	Never	643 (90.2%)
Married	395 (52.7%)	1 h	26 (3.6%)
Cohabitating	206 (27.5%)	>1 h	44 (6.2%)
Missing	2	Missing	39
**Alcohol Use**		**Number of Children**	
Never	378 (50.5%)	0	321 (42.7%)
Yes, before pregnancy	327 (43.7%)	1	273 (36.3%)
Yes, currently	43 (5.7%)	2–5	158 (21.0%)
Missing	4	Missing	0
**BMI (kg/m**^**2**^**)**			
(0, 25]	366 (51.7%)		
(25, 29.9]	212 (29.9%)		
(29.9, 51]	130 (18.4%)		
Missing	0		
**Fetal Sex**			
Female	367 (48.8%)		
Male	385 (51.2%)		
Missing	0		

Distributions of pregnancy geometric mean and visit-specific glyphosate and AMPA concentrations corrected for specific gravity, and visit-specific hormone concentrations, are shown in Table 2 (visit-specific concentrations of AMPA and glyphosate that are not corrected for specific gravity are shown in Table S1). AMPA and glyphosate were detected in at least 70% of samples at all study visits, indicating widespread exposure among participants. Median concentrations of both AMPA and glyphosate were highest at the second study visit, approximately 22 weeks’ gestation. Most hormone concentrations remained relatively stable between visit 1 and visit 3, with the notable exceptions of E3 and progesterone, which increased at visit 3 compared to visit 1 (E3 median: 15.1 ng/mL at visit 1 vs. 39.1 ng/mL at visit 3; progesterone median: 40.6 ng/mL vs. 77.3 ng/mL). These increases are consistent with physiologic endocrine changes across gestation and support the temporal validity of the serum hormone measurements. Intraclass correlation coefficients for all analytes are shown in Table S2, and Pearson correlation coefficients between exposures and outcomes are shown in Table S3; the correlation between glyphosate and AMPA is 0.56.

**Table 2:** Distributions of urinary glyphosate concentrations, corrected for specific gravity, and serum hormone concentrations among 752 women in PROTECT birth cohort in Puerto Rico, 2011–2019.

	Visit	*N*	Min	P25	Median	P75	P90	Max	GM	GSD	IQR
**AMPA (μg/L)**	Avg	752	0.10	0.28	0.41	0.62	0.90	6.31	0.42	1.85	0.34
	1	641	0.09	0.22	0.39	0.63	0.97	4.91	0.39	2.04	0.40
	2	249	0.05	0.36	0.51	0.73	1.21	4.41	0.52	1.93	0.37
	3	465	0.09	0.20	0.41	0.68	1.08	6.31	0.40	2.19	0.48
**Glyphosate (μg/L)**	Avg	752	0.08	0.31	0.49	0.75	1.10	6.07	0.49	1.93	0.44
	1	641	0.09	0.29	0.51	0.81	1.17	6.07	0.49	2.09	0.52
	2	249	0.10	0.38	0.59	0.88	1.36	5.09	0.59	1.98	0.50
	3	465	0.08	0.27	0.47	0.77	1.22	4.28	0.47	2.10	0.51
**CRH (pg/mL)**	1	635	3.47	17.9	47.8	93.2	130	254	39.9	2.77	75.4
	3	465	3.47	18.1	47.7	98.5	141	249	41.3	2.78	80.4
**E3 (mg/mL)**	1	633	0.74	11.3	15.1	22.2	31.7	86.1	15.8	1.73	10.9
	3	465	6.90	29.4	39.1	51.8	65.7	265	39.2	1.58	22.4
**Prog (ng/mL)**	1	634	10.1	29.5	40.6	55.5	73.4	283	40.8	1.60	26.0
	3	465	19.4	52.5	77.3	108	151	1037	77.0	1.72	55.5
**T (pg/mL)**	1	634	2.80	47.4	85.4	519	775	2103	136	3.67	472
	3	465	9.20	55.6	97.2	602	923	2205	155	3.56	546
**TSH (uIU/mL)**	1	629	0.00	0.71	1.11	1.71	2.44	41	1.04	2.25	1.00
	3	463	0.11	0.76	1.21	1.81	2.49	25.7	1.17	1.95	1.06
**FT4 (ng/dL)**	1	633	0.35	0.92	1.02	1.13	1.22	1.72	1.02	1.18	0.21
	3	465	0.44	0.86	0.98	1.09	1.19	1.42	0.97	1.19	0.23
**SHBG (pg/mL)**	1	635	47.6	405	538	650	795	1461	511	1.46	245
	3	465	142	455	581	742	912	1428	578	1.44	287
**T3 (mg/mL)**	1	632	0.11	1.10	1.64	2.03	2.32	4.09	1.42	1.72	0.93
	3	464	0.11	1.23	1.69	2.06	2.34	3.15	1.49	1.66	0.83
**T4 (ug/dL)**	1	632	6.80	10.7	11.9	13.3	14.4	19.0	11.8	1.18	2.60
	3	465	5.30	10.4	11.6	13.1	14.2	18.6	11.6	1.19	2.70

*LOD* limit of detection, *Min* minimum, *Max* maximum, *GM* geometric mean, *GSD* geometric standard deviation, *IQR* interquartile range, *AMPA* aminomethyl phosphonic acid, *CRH* corticotropin releasing hormone, *E3* estriol, *Prog* progesterone, *T* testosterone, *TSH* thyroid stimulating hormone, *FT4* free thyroxine, *SHBG* steroid hormone binding globulin, *T3* total triiodothyronine, *T4* total thyroxine.

The results from mixed models evaluating the percent change in repeated hormone concentrations associated with IQR increases in repeated AMPA and glyphosate concentrations are shown in Table 3. IQR increases in AMPA and glyphosate were associated with 10.6% (95% CI: –18.1, –2.4) and 8.3% (95% CI: –14.6, –1.4) lower E3 concentrations, respectively. Conversely, an IQR increase in AMPA was associated with 4.0% higher T3 (95% CI: 0.9, 7.0). Although not statistically significant at alpha=0.05, an IQR increase in glyphosate was marginally associated with 5.4% lower progesterone (95% CI: –10.6, 0.1) and 1.4% higher fT4 (95% CI: –0.1, 2.8). Exploration of AMPA/GLY concentrations as quartiles did not provide strong evidence of nonlinear associations with hormones (Table S4), though there was a significant association between an increase in AMPA from quartile 1 to quartile 2 and 2.74% reduced T4 (95% CI: -5.26, -0.23).

**Table 3:** Percent change in repeated hormone concentrations per IQR increase in repeated urinary AMPA and glyphosate concentrations in the PROTECT birth cohort in Puerto Rico, 2011–2019.

		AMPA			Glyphosate	
	*N*	%Δ (95% CI)	*P*	*N*	%Δ (95% CI)	*P*
CRH	941	–0.92 (–8.36, 7.12)	0.817	941	3.51 (–2.93, 10.4)	0.293
E3	**940**	**–10.6 (–18.1, –2.38)**	**0.013**	**940**	**–8.26 (–14.6, –1.41)**	**0.020**
Prog	940	–2.87 (–9.33, 4.05)	0.408	940	–5.40 (–10.6, 0.09)	0.055
T	940	–2.35 (–8.25, 3.92)	0.454	940	–0.03 (–5.09, 5.30)	0.991
TSH	933	2.12 (–6.08, 11.0)	0.624	933	0.56 (–6.26, 7.88)	0.876
FT4	939	0.11 (–1.60, 1.83)	0.899	939	1.35 (–0.07, 2.78)	0.064
SHBG	941	–0.43 (–4.21, 3.34)	0.822	941	–0.07 (–3.22, 3.07)	0.963
T3	**937**	**3.98 (0.97, 7.00)**	**0.010**	937	1.58 (–0.93, 4.10)	0.218
T4	938	–0.52 (–2.43, 1.38)	0.593	938	0.56 (–1.04, 2.16)	0.490

N reflects participants with complete data on urinary glyphosate/AMPA concentrations, serum hormone measurements, and all model covariates.

Estimates were derived from LME models adjusted for continuous maternal age and pre-pregnancy BMI, and categorical forms of maternal education and parity, fetal sex, hormone batch measurement method, and specific gravity. Models also included a random intercept for correlations within study participants.

*IQR* interquartile range, *LME* linear mixed effects, *AMPA* aminomethyl phosphonic acid, *%Δ* percent change, *CI* confidence interval, *CRH* corticotropin releasing hormone, *E3* estriol, *Prog* progesterone, *T* testosterone, *TSH* thyroid stimulating hormone, *FT4* free thyroxine, *SHBG* steroid hormone binding globulin, *T3* total triiodothyronine, *T4* total thyroxine.

**Fig. 1:**
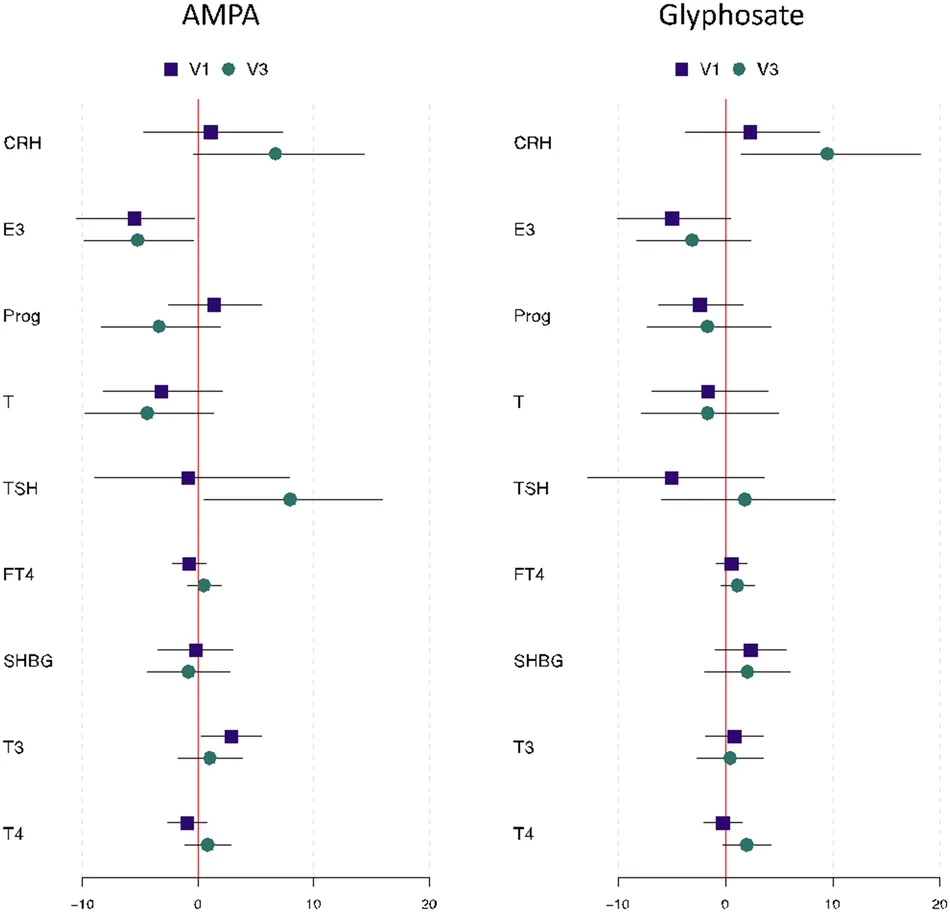
Associations between urinary AMPA and glyphosate concentrations and hormone concentrations by gestational visit. Squares (visit 1) and circles (visit 3) represent percent change (95% confidence interval) in visit-specific hormone concentrations per IQR increase in urinary geometric mean concentrations of AMPA (left panel) and glyphosate (right panel) across pregnancy among 752 women in the PROTECT birth cohort in Puerto Rico, 2011-2019. Models were adjusted for continuous maternal age and pre-pregnancy BMI, and categorical forms of maternal education and parity, fetal sex, and hormone batch measurement method. Pregnancy geometric mean concentrations are corrected for specific gravity.

Results from linear models regressing visit-specific hormone concentrations on geometric mean AMPA and glyphosate concentrations across pregnancy are shown in Fig. 1 (corresponding numerical data is shown in Table S5). An IQR increase in AMPA geometric mean was associated with 5.5% lower E3 (95% CI: –10.5, –0.3) at visit 1, 5.2% lower E3 (95% CI: –9.8, –0.4) at visit 3, and 2.9% higher T3 (95% CI: 0.3, 5.5) at visit 1. Additionally, an IQR increase in AMPA geometric mean was associated with 7.9% higher TSH (95% CI: 0.5, 15.9) at visit 3, while an IQR increase in glyphosate geometric mean was associated with 9.5% higher CRH (95% CI: 1.5, 18.2) at visit 3. Results using visit-specific glyphosate and AMPA concentrations measured concurrently with hormone concentrations at prenatal visits 1 and 3 are presented in Table S6.

Finally, results from LME models with interaction terms for fetal sex are shown in Table S7. While none of the interaction terms were statistically significant, several associations differed in magnitude and significance by fetal sex. The inverse association between AMPA and E3 was stronger, and statistically significant, among participants carrying female fetuses (% change: –16.1; 95% CI: –25.6, –5.5), compared to those carrying male fetuses (% change: –5.2; 95% CI: –15.5, 6.5). Conversely, the positive association between AMPA and T3 was stronger among male fetuses (% change: +5.3; 95% CI: 1.3, 9.3) than female fetuses (% change: +2.67; 95% CI: –1.4, 6.7). Additionally, glyphosate was significantly inversely associated with E3 (% change: –9.3; 95% CI: –17.8, –0.1) and positively associated with fT4 (% change: +2.1; 95% CI: 0.1, 4.0) only among participants carrying female fetuses.

## Discussion

### Summary of main findings

In this study, we evaluated associations between urinary concentrations of glyphosate and its primary degradate AMPA and repeated measures of maternal hormone levels across pregnancy. To our knowledge, this is the first human study to examine how these exposures relate to both sex steroid and thyroid hormones during gestation. We observed consistent inverse associations between both glyphosate and AMPA and E3, a key estrogen involved in placental function and fetal development. These findings were detected across multiple models and time points. AMPA was also associated with elevated T3 throughout pregnancy and higher TSH in later gestation, while glyphosate was linked to higher CRH later in pregnancy. Overall, these findings suggest hormone-specific and temporally sensitive associations, with potential implications for multiple endocrine pathways. While fetal sex did not significantly modify associations, notable trends were observed. Inverse associations between AMPA and E3 were stronger among pregnancies with female fetuses, while positive associations between AMPA and T3 were more pronounced in those with male fetuses, highlighting the possibility of sex-specific endocrine responses.

### Main discussion and contextualization of results

Our findings build on limited human research exploring glyphosate’s endocrine-disrupting potential during pregnancy and align with mechanistic evidence from in vitro and animal studies. Reductions in estriol and suggestive decreases in progesterone are consistent with experimental work demonstrating that glyphosate and glyphosate-based herbicides inhibit aromatase activity and disrupt sex hormone synthesis [21, 22]. These hormonal shifts have been associated with impaired placental function and pregnancy maintenance in rat models [23, 31]. Despite growing concern, epidemiologic data linking glyphosate exposure to sex steroid disruption in human pregnancy have been virtually absent; this study begins to address that gap.

Our results also complement prior studies reporting adverse reproductive outcomes associated with glyphosate exposure. For instance, previous analyses within the PROTECT cohort have found associations between prenatal urinary glyphosate and AMPA concentrations and hormonally-regulated outcomes, such as increased odds of preterm birth and neurodevelopmental differences in early childhood, including altered cognitive and behavioral outcomes [5, 6]. In addition, Gerona et al. (2022) observed reduced fetal growth linked to higher first-trimester glyphosate levels [15]. While that study did not evaluate hormone concentrations, our findings raise the possibility that reductions in placental estrogens like E3 could mediate such effects. Similarly, observed increases in T3 and TSH, particularly in relation to AMPA, align with animal data indicating that glyphosate exposure may dysregulate thyroid hormone feedback loops [26]. These shifts are consistent with disruption at the level of central hormonal regulation (i.e., altered signaling between the hypothalamus and pituitary) rather than dysfunction originating in the thyroid gland itself. Such central dysregulation may reflect feedback alterations in the hypothalamic-pituitary-thyroid axis, which governs thyroid hormone production and is particularly sensitive to environmental stressors during pregnancy.

The association between glyphosate and elevated CRH concentrations in late pregnancy could potentially reflect perturbation of the hypothalamic-pituitary-adrenal axis. Animal studies suggest that glyphosate impairs adrenal steroidogenesis and reduces ACTH and corticosterone levels, which could lead to compensatory increases in CRH [28]. Elevated CRH has been linked to shortened gestation and increased risk of preterm birth, pointing to possible clinical relevance of these endocrine changes [32, 33]. Notably, previous analyses within the PROTECT cohort observed that associations between glyphosate exposure and preterm birth were strongest for spontaneous preterm birth, particularly when glyphosate was measured at the third study visit [5]. This pattern may be consistent with our finding of elevated CRH concentrations associated with glyphosate at visit 3, given CRH’s known role in initiating parturition.

While fetal sex did not statistically modify associations, models with sex*exposure interaction terms revealed consistent trends: inverse associations between AMPA and E3 were stronger among pregnancies with female fetuses, while AMPA was more strongly associated with T3 in pregnancies with male fetuses. These patterns echo previous findings of sex-specific susceptibility to environmental exposures and support the need to explore fetal sex as a potential effect modifier in endocrine-related studies [34]. Developmental exposure to endocrine-disrupting chemicals has been shown to produce sex-specific effects on neurodevelopment and hypothalamic differentiation beginning in early fetal life, and animal studies have consistently demonstrated that such exposures can lead to differential behavioral and hormonal outcomes depending on sex [35, 36]. These sex-specific trends not only reinforce the importance of considering fetal sex in endocrine research but also point to the need for mechanistic insight into how glyphosate and AMPA may differentially affect hormonal pathways.

Mechanistically, the hormonal changes observed in this study are plausible. Glyphosate and GBHs have been shown to inhibit aromatase, reduce estrogen and progesterone synthesis, and alter endocrine signaling in reproductive tissues [21, 23]. Glyphosate has also been linked to abnormal pituitary-thyroid signaling, with evidence of TSH dysregulation in animals despite normal T3 and T4 levels [26]. In the context of HPA axis disruption, glyphosate exposure has been associated with reduced adrenal output and altered feedback regulation, raising concern about stress hormone imbalances and CRH overproduction [28].

Beyond direct hormonal mechanisms, indirect pathways may also contribute. Oxidative stress and mitochondrial dysfunction have been observed in placental and ovarian tissues following glyphosate exposure, potentially impairing hormone synthesis and placental function [23, 37]. Co-formulants in commercial GBHs, such as polyethoxylated tallow amine (POEA), may also independently disrupt endocrine signaling and amplify glyphosate’s toxicity [22, 38]. These synergistic effects may enhance biologic activity beyond what is observed with glyphosate alone.

Sensitivity analyses revealed temporal variation in associations. Reductions in E3 were detected at both early and late gestational time points, while associations with thyroid-related hormones were more prominent later in pregnancy. These patterns suggest that mid-to-late gestation may represent a critical window of susceptibility, particularly given the concurrent activation of placental, fetal, and maternal endocrine systems. This is consistent with prior research indicating that hormonal trajectories and their vulnerability to environmental exposures vary by gestational timing [16, 39]. For instance, thyroid hormone levels, especially T3 and TSH, increase substantially during the second and third trimesters to meet fetal metabolic demands, and are therefore more likely to reveal disruptions during these periods. Similarly, estrogen production surges in late gestation in support of uterine growth and parturition. Our ability to detect these temporally distinct relationships reflects a strength of this study’s repeated-measures design, which is well-suited to capturing dynamic hormonal shifts over the course of pregnancy.

As previously described, urinary glyphosate and AMPA detection frequencies among PROTECT participants fall within 51-88%, lower than those reported in some other pregnancy cohorts [5, 6]. These differences may, in part, reflect variation in analytical methods. For example, the TIDES study, a multi-center U.S. birth cohort, reported detectable urinary glyphosate and AMPA concentrations in over 90% of pregnant participants, but reported lower limits of detection (LOD) than the present study (0.014 and 0.013 µg/L for glyphosate and AMPA, respectively, compared with 0.2 µg/L for both in PROTECT) [8]. Despite these lower LODs, median concentrations of both glyphosate and AMPA in TIDES were lower than those observed in PROTECT (median[IQR]: 0.22 [0.51] µg/L and 0.14 [0.20] µg/L for glyphosate and AMPA, respectively), suggesting that higher detectability does not necessarily correspond to higher exposure levels. By comparison, data from the 2015-2016 National Health and Nutrition Examination Survey, which used the same LOD as PROTECT, reflect detectable glyphosate concentrations in approximately 66% of participants, with a median (IQR) urinary concentration of 0.29 (0.38) µg/L, also substantially lower than concentrations observed in PROTECT [6]. Taken together, these comparisons suggest that background glyphosate exposure in the PROTECT cohort is comparable to or modestly higher than that observed in the general U.S. population. Regional differences in agricultural practices, dietary patterns, and timing of urine collection should be considered when assessing the generalizability of these findings to other populations.

While these comparisons help contextualize exposure levels, they do not identify specific sources of glyphosate and AMPA exposure in this population, which are unclear. The study region is largely rural and relies on the northern karst aquifer system, which is highly vulnerable to contamination and supplies drinking water for PROTECT participants [40, 41]. In the absence of extensive large-scale agriculture in Puerto Rico, additional potential exposure pathways, inferred from studies in other populations, may include contaminated drinking water, dietary sources, or residential and municipal pesticide use; however, there are no published data directly characterizing these sources in this setting [7, 13, 42–44].

This study is strengthened by its prospective design, large sample size, and repeated measurements of both exposures and hormonal outcomes across multiple endocrine axes, which enabled the examination of temporal patterns and potential windows of susceptibility. By integrating robust exposure assessment, detailed hormone profiling, and a prospective study design, this research provides preliminary evidence on how glyphosate and AMPA exposures may relate to maternal endocrine function during pregnancy. Findings from this study inform our understanding of health effects related to glyphosate exposure, demonstrate the need for further research in this area, and may contribute to policy and regulatory discussions surrounding the use of GBHs in environments where pregnant individuals may be disproportionately exposed.

However, certain limitations warrant consideration. As with all observational research, causal inference is inherently limited, and residual confounding may remain despite careful adjustment for key covariates. Although batch-related differences in hormone assays were accounted for analytically, some measurement variability may persist. Additionally, sample sizes were larger at the first study visit (roughly 600) than the third (roughly 400), which may influence power and precision of visit-specific estimates. Serum hormone levels, while informative, do not capture receptor-level activity or tissue-specific signaling. Similarly, urinary glyphosate and AMPA concentrations reflect short-term exposure and may not fully characterize cumulative burden across pregnancy, given elimination half-lives ranging between 9 and 24 h [45–48]. Lastly, while prior analyses in the PROTECT cohort have explored associations with preterm birth and early neurodevelopmental outcomes, we did not evaluate these downstream endpoints in the present study. Nonetheless, this study offers insights into how widely prevalent environmental exposures may impact endocrine function in human pregnancy. Future research should continue to assess these outcomes in larger samples with repeated exposure and outcome measurements across multiple windows of pregnancy, including the first and third trimesters, to better characterize potential window-specific associations. Studies should also examine dysregulation of hormones as a mediator by which glyphosate exposure may impact maternal and child health, incorporate longitudinal follow-up of child outcomes, and explore mechanistic pathways at the placental and molecular level, while expanding assessments across diverse populations to improve generalizability and policy relevance.

### Conclusion

This study is the first to document associations between urinary glyphosate and AMPA concentrations and multiple hormonal pathways during human pregnancy. By identifying potential disruptions in sex steroid, thyroid, and stress hormone axes, our findings contribute critical new evidence to a limited field and highlight the importance of endocrine-sensitive windows in gestation. As concerns grow over the health implications of pervasive environmental exposures, this research supports the need for precautionary measures to protect maternal and child health during critical periods of development.

## Supplementary information

Supplementary tables

## Data Availability

Data are available from the corresponding author on reasonable request.
