# Supplementary material for: Glyphosate exposure and hormonal disruption in pregnancy: evidence from a birth cohort in Puerto Rico
Source: J Expo Sci Environ Epidemiol. 2026 Jun 15;36(6):1205–12. doi: 10.1038/s41370-026-00902-6 (PMC13727658; doi:10.1038/s41370-026-00902-6)
Supplement: Supplementary file 1 [file 41370_2026_902_MOESM1_ESM.docx]

**Table S1.** Distributions of raw urinary glyphosate exposure biomarkers and serum hormone concentrations, in µg/L, among 752 women in PROTECT birth cohort in Puerto Rico, 2011-2019.

|  | **Visit** | **N** | **%>LOD^1^** | **P25** | **Median** | **P75** | **P90** | **Max** | **GM** | **GSD** | **IQR** |
| --- | --- | --- | --- | --- | --- | --- | --- | --- | --- | --- | --- |
| **AMPA** | 1 | 660 | 72.1% | 0.14 | 0.36 | 0.67 | 1.10 | 6.71 | 0.37 | 2.23 | 0.52 |
|  | 2 | 251 | 87.3% | 0.30 | 0.50 | 0.75 | 1.16 | 5.34 | 0.48 | 2.13 | 0.45 |
|  | 3 | 468 | 68.8% | 0.14 | 0.34 | 0.63 | 1.07 | 7.97 | 0.36 | 2.27 | 0.49 |
| **Glyphosate** | 1 | 660 | 80.2% | 0.25 | 0.50 | 0.88 | 1.33 | 4.14 | 0.47 | 2.29 | 0.63 |
|  | 2 | 251 | 85.7% | 0.33 | 0.58 | 0.94 | 1.50 | 6.69 | 0.54 | 2.23 | 0.61 |
|  | 3 | 468 | 76.1% | 0.22 | 0.43 | 0.73 | 1.27 | 5.41 | 0.42 | 2.29 | 0.51 |

^1^ LOD for GLY and AMPA is 0.2 µg/L.

**Table S2.** Intraclass correlation coefficients of urinary glyphosate exposure biomarkers and serum hormone concentrations across prenatal visits in the PROTECT birth cohort in Puerto Rico, 2011-2019.

|  | **ICC** | **Low CI** | **High CI** |
| --- | --- | --- | --- |
| **AMPA** | 0.241 | 0.112 | 0.351 |
| **Glyphosate** | 0.348 | 0.232 | 0.445 |
| **CRH** | 0.631 | 0.558 | 0.690 |
| **E3** | -0.083 | -0.243 | 0.059 |
| **Prog** | 0.165 | 0.027 | 0.283 |
| **T** | 0.814 | 0.775 | 0.845 |
| **TSH** | 0.763 | 0.714 | 0.803 |
| **FT4** | 0.628 | 0.554 | 0.688 |
| **SHBG** | 0.767 | 0.719 | 0.806 |
| **T3** | 0.717 | 0.660 | 0.764 |
| **T4** | 0.747 | 0.695 | 0.789 |

AMPA: aminomethylphosphonic acid; ICC: Intraclass correlation coefficient; CI: confidence interval; CRH: corticotropin releasing hormone; E3: estriol; Prog: progesterone; T: testosterone; TSH: thyroid stimulating hormone; FT4: free thyroxine; SHBG: steroid hormone binding globulin; T3: total triiodothyronine; T4: total thyroxine.

**Table S3.** Pearson correlation coefficients between urinary glyphosate biomarkers and serum hormone concentrations in the PROTECT birth cohort in Puerto Rico, 2011-2019. Bottom triangle shows prenatal visit 1 coefficients and top triangle shows prenatal visit 3 coefficients. Log-normal variables were transformed.

|  | **AMPA** | **Glyphosate** | **CRH** | **E3** | **Prog** | **T** | **TSH** | **FT4** | **SHBG** | **T3** | **T4** |
| --- | --- | --- | --- | --- | --- | --- | --- | --- | --- | --- | --- |
| **AMPA** |  | 0.550 | 0.092 | -0.082 | 0.028 | -0.125 | 0.101 | 0.118 | 0.074 | 0.092 | 0.068 |
| **Glyphosate** | 0.555 |  | 0.046 | -0.071 | -0.004 | -0.041 | -0.008 | 0.102 | 0.041 | 0.032 | 0.128 |
| **CRH** | 0.042 | 0.070 |  | 0.333 | 0.473 | -0.716 | 0.324 | 0.529 | 0.429 | 0.539 | 0.001 |
| **E3** | -0.062 | -0.028 | 0.288 |  | 0.599 | -0.276 | 0.226 | 0.195 | 0.425 | 0.248 | -0.057 |
| **Prog** | -0.007 | -0.030 | 0.437 | 0.507 |  | -0.418 | 0.257 | 0.333 | 0.528 | 0.326 | 0.053 |
| **T** | -0.061 | -0.066 | -0.762 | -0.312 | -0.451 |  | -0.359 | -0.594 | -0.433 | -0.660 | -0.082 |
| **TSH** | 0.001 | 0.001 | 0.187 | 0.188 | 0.145 | -0.237 |  | 0.260 | 0.280 | 0.392 | -0.013 |
| **FT4** | 0.052 | 0.088 | 0.406 | 0.112 | 0.280 | -0.494 | 0.001 |  | 0.405 | 0.489 | 0.475 |
| **SHBG** | -0.038 | 0.038 | 0.387 | 0.371 | 0.461 | -0.417 | 0.083 | 0.302 |  | 0.242 | 0.031 |
| **T3** | 0.158 | 0.100 | 0.548 | 0.261 | 0.384 | -0.672 | 0.217 | 0.431 | 0.233 |  | 0.206 |
| **T4** | -0.005 | 0.038 | -0.055 | 0.032 | 0.049 | 0.020 | -0.101 | 0.509 | 0.081 | 0.134 |  |

AMPA: aminomethylphosphonic acid; CRH: corticotropin releasing hormone; E3: estriol; Prog: progesterone; T: testosterone; TSH: thyroid stimulating hormone; FT4: free thyroxine; SHBG: steroid hormone binding globulin; T3: total triiodothyronine; T4: total thyroxine.

**Table S4.** Percent change in repeated hormones with a change from the lowest quartile of repeated exposures in the PROTECT birth cohort in Puerto Rico, 2011-2019. Estimates were derived from LME models adjusted for continuous maternal age and pre-pregnancy BMI, and categorical forms of maternal education and parity, fetal sex, hormone batch measurement method, and specific gravity. Models also included a random intercept for correlations within study participants.

|  | **AMPA** | | | | | **Glyphosate** | | | | |
| --- | --- | --- | --- | --- | --- | --- | --- | --- | --- | --- |
|  | **N group** | **% Change** | **Low CI** | **Hi CI** | **P** | **N group** | **% Change** | **Low CI** | **Hi CI** | **P** |
| CRH q2 | 196 | 4.02 | -6.59 | 15.84 | 0.473 | 250 | 2.59 | -7.75 | 14.09 | 0.637 |
| CRH q3 | 248 | -2.35 | -11.90 | 8.23 | 0.651 | 249 | 3.56 | -7.30 | 15.70 | 0.536 |
| CRH q4 | 250 | -1.70 | -12.04 | 9.85 | 0.762 | 249 | 5.93 | -5.85 | 19.19 | 0.339 |
| E3 q2 | 196 | 4.34 | -7.60 | 17.82 | 0.494 | 250 | 1.59 | -9.88 | 14.53 | 0.796 |
| E3 q3 | 248 | -3.21 | -13.77 | 8.64 | 0.580 | **249** | **-11.70** | **-22.11** | **0.09** | **0.053** |
| E3 q4 | **250** | **-15.58** | **-25.48** | **-4.37** | **0.008** | **249** | **-12.95** | **-23.75** | **-0.63** | **0.041** |
| Prog q2 | 196 | 1.81 | -7.47 | 12.02 | 0.713 | 250 | 1.01 | -8.05 | 10.96 | 0.834 |
| Prog q3 | 248 | -1.78 | -10.31 | 7.56 | 0.699 | 249 | -4.83 | -13.74 | 5.00 | 0.324 |
| Prog q4 | 250 | -3.96 | -12.93 | 5.94 | 0.420 | 249 | -9.09 | -18.05 | 0.86 | 0.073 |
| T q2 | 196 | 1.91 | -6.19 | 10.70 | 0.655 | 250 | 7.51 | -0.99 | 16.75 | 0.086 |
| T q3 | 248 | 4.29 | -3.82 | 13.08 | 0.310 | 249 | 3.09 | -5.42 | 12.37 | 0.489 |
| T q4 | 250 | -2.59 | -10.85 | 6.44 | 0.563 | 249 | 2.82 | -6.43 | 12.98 | 0.563 |
| TSH q2 | 196 | 2.92 | -7.86 | 14.96 | 0.610 | 250 | 0.61 | -9.91 | 12.36 | 0.914 |
| TSH q3 | 248 | -1.18 | -11.34 | 10.14 | 0.830 | 249 | -4.03 | -14.50 | 7.72 | 0.485 |
| TSH q4 | 250 | 1.10 | -10.26 | 13.91 | 0.857 | 249 | 1.33 | -10.80 | 15.11 | 0.840 |
| FT4 q2 | 196 | -0.98 | -3.29 | 1.33 | 0.408 | 250 | 1.15 | -1.13 | 3.44 | 0.324 |
| FT4 q3 | 248 | 0.70 | -1.54 | 2.94 | 0.543 | 249 | 2.02 | -0.36 | 4.41 | 0.098 |
| FT4 q4 | 250 | 0.17 | -2.28 | 2.61 | 0.895 | 249 | 2.11 | -0.49 | 4.70 | 0.113 |
| SHBG q2 | 196 | -1.31 | -6.33 | 3.72 | 0.611 | 250 | -1.93 | -6.94 | 3.08 | 0.451 |
| SHBG q3 | 248 | -2.52 | -7.43 | 2.39 | 0.316 | 249 | -0.16 | -5.40 | 5.07 | 0.952 |
| SHBG q4 | 250 | -0.16 | -5.54 | 5.22 | 0.954 | 249 | -2.36 | -8.08 | 3.36 | 0.420 |
| T3 q2 | 196 | -0.09 | -4.18 | 3.99 | 0.964 | 250 | 0.76 | -3.27 | 4.80 | 0.711 |
| T3 q3 | 248 | 2.34 | -1.61 | 6.29 | 0.247 | 249 | 3.08 | -1.13 | 7.30 | 0.153 |
| T3 q4 | **250** | **4.44** | **0.13** | **8.75** | **0.044** | 249 | 3.55 | -1.03 | 8.12 | 0.130 |
| T4 q2 | **196** | **-2.74** | **-5.26** | **-0.23** | **0.033** | 250 | -0.73 | -3.24 | 1.77 | 0.567 |
| T4 q3 | 248 | -1.67 | -4.13 | 0.79 | 0.185 | 249 | -0.12 | -2.74 | 2.50 | 0.929 |
| T4 q4 | 250 | -0.42 | -3.14 | 2.29 | 0.759 | 249 | 0.52 | -2.38 | 3.42 | 0.726 |

LME: linear mixed effects; AMPA: aminomethylphosphonic acid; CI: confidence interval; CRH: corticotropin releasing hormone; E3: estriol; Prog: progesterone; T: testosterone; TSH: thyroid stimulating hormone; FT4: free thyroxine; SHBG: steroid hormone binding globulin; T3: total triiodothyronine; T4: total thyroxine.

**Table S5**. Percent change in visit-specific hormones per IQR increase in pregnancy average exposures in the PROTECT birth cohort in Puerto Rico, 2011-2019. Models were adjusted for continuous maternal age and pre-pregnancy BMI, and categorical forms of maternal education and parity, fetal sex, and hormone batch measurement method.

|  |  | AMPA |  |  |  | Glyphosate |  |
| --- | --- | --- | --- | --- | --- | --- | --- |
| Visit 1 | N | %Δ (95% CI) | P |  | N | %Δ (95% CI) | P |
| CRH | 598 | 1.10 (-4.74, 7.31) | 0.718 |  | 598 | 2.32 (-3.75, 8.76) | 0.463 |
| E3 | **597** | **-5.52 (-10.5, -0.28)** | **0.040** |  | 597 | -4.96 (-10.1, 0.45) | 0.072 |
| Prog | 597 | 1.38 (-2.55, 5.46) | 0.497 |  | 597 | -2.39 (-6.26, 1.64) | 0.242 |
| T | 597 | -3.20 (-8.24, 2.11) | 0.233 |  | 597 | -1.60 (-6.85, 3.95) | 0.565 |
| TSH | 592 | -0.87 (-8.94, 7.91) | 0.840 |  | 592 | -4.98 (-12.9, 3.60) | 0.247 |
| FT4 | 596 | -0.77 (-2.21, 0.67) | 0.295 |  | 596 | 0.58 (-0.90, 2.05) | 0.445 |
| SHBG | 598 | -0.20 (-3.44, 3.03) | 0.901 |  | 598 | 2.37 (-0.95, 5.68) | 0.162 |
| T3 | **595** | **2.86 (0.25, 5.47)** | **0.032** |  | 595 | 0.82 (-1.86, 3.50) | 0.549 |
| T4 | 595 | -0.94 (-2.68, 0.79) | 0.286 |  | 595 | -0.25 (-2.02, 1.53) | 0.786 |
|  |  | AMPA |  |  |  | Glyphosate |  |
| Visit 3 | N | %Δ (95% CI) | P |  | N | %Δ (95% CI) | P |
| CRH | 435 | 6.70 (-0.42, 14.3) | 0.066 |  | **435** | **9.53 (1.50, 18.2)** | **0.020** |
| E3 | **435** | **-5.24 (-9.83, -0.41)** | **0.034** |  | 435 | -3.09 (-8.29, 2.41) | 0.266 |
| Prog | 435 | -3.37 (-8.36, 1.88) | 0.205 |  | 435 | -1.66 (-7.27, 4.28) | 0.576 |
| T | 435 | -4.40 (-9.82, 1.35) | 0.132 |  | 435 | -1.63 (-7.79, 4.94) | 0.619 |
| TSH | **433** | **7.95 (0.51, 15.9)** | **0.036** |  | 433 | 1.84 (-5.93, 10.3) | 0.652 |
| FT4 | 435 | 0.54 (-0.88, 1.97) | 0.456 |  | 435 | 1.15 (-0.42, 2.73) | 0.151 |
| SHBG | 435 | -0.81 (-4.38, 2.76) | 0.658 |  | 435 | 2.05 (-1.89, 6.00) | 0.308 |
| T3 | 434 | 1.04 (-1.76, 3.84) | 0.465 |  | 434 | 0.44 (-2.65, 3.54) | 0.779 |
| T4 | 435 | 0.85 (-1.15, 2.85) | 0.404 |  | 435 | 1.99 (-0.21, 4.19) | 0.077 |

AMPA: aminomethylphosphonic acid; %Δ: percent change; CI: confidence interval; CRH: corticotropin releasing hormone; E3: estriol; Prog: progesterone; T: testosterone; TSH: thyroid stimulating hormone; FT4: free thyroxine; SHBG: steroid hormone binding globulin; T3: total triiodothyronine; T4: total thyroxine.

**Table S6.** Percent change in visit-specific hormones per IQR increase in visit-specific exposures in the PROTECT birth cohort in Puerto Rico, 2011-2019. Models were adjusted for continuous maternal age, pre-pregnancy BMI, and urine specific gravity, and categorical forms of maternal education and parity, fetal sex, and hormone batch measurement method.

***Visit 1***

|  |  | AMPA |  |  |  | Glyphosate |  |
| --- | --- | --- | --- | --- | --- | --- | --- |
|  | N | %Δ (95% CI) | P |  | N | %Δ (95% CI) | P |
| CRH | 574 | -5.09 (-14.43, 5.27) | 0.324 |  | 574 | 0.36 (-7.53, 8.94) | 0.931 |
| E3 | 573 | -8.40 (-16.44, 0.41) | 0.062 |  | 573 | -5.86 (-12.45, 1.22) | 0.103 |
| Prog | 573 | -1.09 (-7.64, 5.91) | 0.753 |  | 573 | -4.53 (-9.54, 0.76) | 0.092 |
| T | 573 | -0.94 (-9.69, 8.64) | 0.841 |  | 573 | -1.00 (-7.96, 6.49) | 0.787 |
| TSH | 568 | -0.27 (-13.98, 15.63) | 0.971 |  | 568 | -0.44 (-11.37, 11.84) | 0.941 |
| FT4 | 572 | -0.30 (-2.81, 2.21) | 0.814 |  | 572 | 1.38 (-0.61, 3.36) | 0.174 |
| SHBG | 574 | -3.49 (-9.07, 2.08) | 0.220 |  | 574 | 1.49 (-2.92, 5.89) | 0.509 |
| T3 | **571** | **8.46 (3.88, 13.03)** | **0.000** |  | 571 | 2.37 (-1.29, 6.02) | 0.205 |
| T4 | 571 | -1.02 (-4.03, 1.98) | 0.505 |  | 571 | 0.50 (-1.87, 2.87) | 0.680 |

***Visit 3***

|  |  | AMPA |  |  |  | Glyphosate |  |
| --- | --- | --- | --- | --- | --- | --- | --- |
|  | N | %Δ (95% CI) | P |  | N | %Δ (95% CI) | P |
| CRH | 367 | 3.75 (-7.53, 16.40) | 0.531 |  | 367 | 9.42 (-1.69, 21.79) | 0.100 |
| E3 | **367** | **-9.01 (-16.19, -1.22)** | **0.025** |  | 367 | -5.54 (-12.54, 2.03) | 0.148 |
| Prog | 367 | -1.80 (-10.28, 7.49) | 0.694 |  | 367 | -1.88 (-9.82, 6.76) | 0.660 |
| T | 367 | -9.02 (-17.54, 0.39) | 0.061 |  | 367 | -5.11 (-13.47, 4.05) | 0.265 |
| TSH | 365 | 7.92 (-3.77, 21.04) | 0.193 |  | 365 | -4.32 (-14.07, 6.54) | 0.421 |
| FT4 | 367 | 1.21 (-1.22, 3.64) | 0.329 |  | 367 | 2.08 (-0.18, 4.34) | 0.072 |
| SHBG | 367 | 1.53 (-4.26, 7.33) | 0.604 |  | 367 | 1.70 (-3.71, 7.11) | 0.538 |
| T3 | 366 | 1.19 (-3.25, 5.64) | 0.600 |  | 366 | -0.03 (-4.18, 4.12) | 0.989 |
| T4 | 367 | 1.53 (-1.87, 4.93) | 0.379 |  | 367 | 2.33 (-0.84, 5.50) | 0.151 |

AMPA: aminomethyl phosphonic acid; %Δ: percent change; CI: confidence interval; CRH: corticotropin releasing hormone; E3: estriol; Prog: progesterone; T: testosterone; TSH: thyroid stimulating hormone; FT4: free thyroxine; SHBG: steroid hormone binding globulin; T3: total triiodothyronine; T4: total thyroxine.

**Table S7**. Percent change in repeated hormones per IQR increase in repeated exposures, by fetal sex, in the PROTECT birth cohort in Puerto Rico, 2011-2019. Models were adjusted for continuous maternal age and pre-pregnancy BMI, and categorical forms of maternal education and parity, specific gravity, and hormone batch measurement method. Models included an interaction term between the exposure variable and an indicator for fetal sex to derive interaction term p-values.

|  |  |  |  | AMPA |  |  |  |
| --- | --- | --- | --- | --- | --- | --- | --- |
|  |  | Females |  |  |  | Males |  |
|  | N | %Δ (95% CI) | P | P-Int | N | %Δ (95% CI) | P |
| CRH | 941 | 1.31 (-8.88, 12.64) | 0.810 | 0.543 | 941 | -2.97 (-12.5, 7.57) | 0.567 |
| E3 | **940** | **-16.1 (-25.6, -5.46)** | **0.004** | 0.125 | 940 | -5.15 (-15.5, 6.45) | 0.370 |
| Prog | 940 | -5.98 (-14.4, 3.27) | 0.199 | 0.319 | 940 | 0.09 (-8.58, 9.58) | 0.984 |
| T | 940 | -2.42 (-10., 6.04) | 0.565 | 0.983 | 940 | -2.30 (-10.2, 6.30) | 0.590 |
| TSH | 933 | -4.25 (-14.3, 6.96) | 0.443 | 0.084 | 933 | 9.40 (-2.40, 22.6) | 0.124 |
| FT4 | 939 | 1.29 (-1.01, 3.59) | 0.273 | 0.133 | 939 | -1.07 (-3.37, 1.23) | 0.363 |
| SHBG | 941 | 1.84 (-3.18, 6.86) | 0.473 | 0.183 | 941 | -2.76 (-7.86, 2.34) | 0.290 |
| T3 | 937 | 2.67 (-1.38, 6.71) | 0.198 | 0.341 | **937** | **5.29 (1.25, 9.33)** | **0.011** |
| T4 | 938 | -0.38 (-2.91, 2.14) | 0.767 | 0.871 | 938 | -0.67 (-3.27, 1.94) | 0.617 |
|  |  |  |  | Glyphosate |  |  |  |
|  |  | Females |  |  |  | Males |  |
|  | N | %Δ (95% CI) | P | P-Int | N | %Δ (95% CI) | P |
| CRH | 941 | 6.48 (-2.46, 16.3) | 0.162 | 0.355 | 941 | 0.96 (-7.10, 9.72) | 0.823 |
| E3 | **940** | **-9.33 (-17.8, 0.05)** | **0.052** | 0.733 | 940 | -7.31 (-15.6, 1.76) | 0.112 |
| Prog | 940 | -4.30 (-11.4, 3.37) | 0.265 | 0.666 | 940 | -6.36 (-13.0, 0.74) | 0.079 |
| T | 940 | -0.26 (-7.08, 7.06) | 0.942 | 0.925 | 940 | 0.18 (-6.37, 7.19) | 0.959 |
| TSH | 933 | -1.98 (-11.0, 7.90) | 0.683 | 0.444 | 933 | 2.89 (-6.10, 12.7) | 0.542 |
| FT4 | **939** | **2.07 (0.12, 4.02)** | **0.038** | 0.290 | 939 | 0.72 (-1.13, 2.57) | 0.448 |
| SHBG | 941 | 0.25 (-4.04, 4.55) | 0.908 | 0.826 | 941 | -0.37 (-4.47, 3.73) | 0.860 |
| T3 | 937 | 0.48 (-2.95, 3.92) | 0.782 | 0.357 | 937 | 2.56 (-0.70, 5.82) | 0.125 |
| T4 | 938 | -0.43 (-2.61, 1.75) | 0.701 | 0.193 | 938 | 1.45 (-0.63, 3.53) | 0.174 |

IQR: Interquartile range; AMPA: aminomethylphosphonic acid; %Δ: percent change; CI: confidence interval; CRH: corticotropin releasing hormone; E3: estriol; Prog: progesterone; T: testosterone; TSH: thyroid stimulating hormone; FT4: free thyroxine; SHBG: steroid hormone binding globulin; T3: total triiodothyronine; T4: total thyroxine.
